# The Story of the Insane from Year to Year

**Published:** 1904-04-23

**Authors:** 


### THE STORY OF THE INSANE FROM YEAR TO YEAR.

Sixteenth Annual Series.

### DORSET COUNTY ASYLUM.

During the year 1902 there were 130 first admissions, 26 readmissions, 38 recoveries, 45 discharged not improved, and 60 died. On the first of January the asylum contained 736 patients, and by the end of the year the number had increased by 10. The total number under treatment during the year was 882, and the average daily number resident was 737. The recoveries stand at 33 per cent. of the admissions, and the deaths at 6 72 on the average number resident. Both of these percentages are lower than what are usually met with in county asylums. Of the year's deaths 17 were caused by cerebral and spinal diseases, 11 by pneumonia and 4 by consumption. Of the year's admissions the assigned cause of insanity was in 23 cases domestic trouble, mental worry, and other moral causes, while among the physical causes hereditary influence accounts for 37, previous attacks for 21, various bodily diseases for 24, and intemperance in drink for 2 only. The admission rate at this asylum has been higher than usual, and Dr. Macdonald thinks that cases which only need ordinary attention and nursing should be kept at their own homes or in the workhouse, and so they should; but for some unknown reason the boarding out system for pauper lunatics has never been popular in England, though it answers admirably in Scotland, and the capitation grant militates directly against their retention in the workhouse. We notice that the weekly cost of maintenance in this asylum is 9s. per head, and of this sum the guardians recover back 4s., which leaves them only 5s. a week to pay for each lunatic sent from their union. If establishment charges be added to the workhouse rates, it would probably be found that the guardians actually save money by sending their chronic cases to the asylum, and certainly the workhouse officials are saved all the trouble of looking after these unwelcome guests. Unfortunately the asylum wards are crowded by these cases, and addition after addition has to be made to accommodate them. It has been more than once pointed out in these columns that very inexpensive improvements to the workhouse wards coupled with a 2s. grant for every lunatic in the workhouse would very likely have the result so much desired by Dr. Macdonald and his *confrères*. Again we are in full accord with him in what he says about criminal lunatics. It is a mystery to us why our Visiting Commitees have borne this "dumping" of jailbirds in their asylums for so many years without even a show of passive resistance. We regret to hear that proper asylum treatment is more delayed by the educated classes than it is by the ordinary patients of our county asylums, and Dr. Macdonald has better grounds of forming an opinion on this subject than most medical superintendents, for his asylum contains 117 private patients; these pay from 10s. to 42s. a week.

